# Microgels of N-Isopropylacrylamide Copolymerized with an Amphiphilic Acid for the Delivery of Doxorubicin

**DOI:** 10.3390/gels10120806

**Published:** 2024-12-07

**Authors:** Teresa G. Rodriguez-Tellez, Héctor Magaña, José M. Cornejo-Bravo, Giovanni Palomino-Vizcaino, Kenia Palomino-Vizcaino

**Affiliations:** 1 Faculty of Chemical Sciences and Engineering, Autonomous University of Baja California, University Boulevard No. 14418, Otay Mesa, Tijuana 22390, Mexico; teresa.rodriguez.tellez@uabc.edu.mx (T.G.R.-T.); hector.magana@uabc.edu.mx (H.M.); jmcornejo@uabc.edu.mx (J.M.C.-B.); 2 Faculty of Health Sciences, Autonomous University of Baja California, University Boulevard No. 1000, Tijuana 22260, Mexico; gpalomino@uabc.edu.mx

**Keywords:** microgels, poly N-isopropylacrylamide, stimuli responsive materials, doxorubicin

## Abstract

This study aims to design microgels that are thermo- and pH-sensitive for controlled doxorubicin (Dox) release in response to tumor microenvironment changes. N-isopropylacrylamide (NIPAAm) is widely used for thermoresponsive tumor-targeted drug delivery systems for the release of therapeutic payloads in response to temperature changes. Herein, a NIPAAm microgel (MP) that is responsive to temperature and pH was designed for the smart delivery of Dox. MP was made from NIPAAm, and polyethylene glycol methyl ether methacrylate (PEGMA) was copolymerized with 5%, 10%, or 15% mol of methacryloylamido hexanoic acid, (CAM5) an amphiphilic acid. We characterized the microgels using FTIR-ATR, DLS, and FESEM. The MP 10% CAM5 exhibited a particle size of 268 nm, with a transition temperature of 44 °C. MP had a drug loading capacity of 13% and entrapment efficiency of 87%. Nearly 100% of the Dox was released at pH 5 and 42 °C, compared to 30% at pH 7.4 and 37 °C. MP 10% CAM5 showed cytocompatibility in HeLa cells using the MTT assay. However, the cell viability assay showed that dox-MP was twice as effective as free Dox. Specifically, 3 μg/mL of free Dox resulted in 74% cell viability, while the same doses of Dox in NP reduced it to 35%. These results are promising for the future tumor-targeted delivery of antineoplastic-drugs, as they may reduce the side effects of Dox.

## 1. Introduction

Cancer encompasses about 80 diseases characterized by abnormal proliferation of cells, that escape to programmed cell death, forming an abnormal cell mass or tumor, except for hematological cancers [1]. The International Agency for Research on Cancer (IARC) estimates 18.1 million new cases and 9.6 million cancer deaths worldwide [2]. It estimates that one in five men and one in six women worldwide will develop cancer in their lifetime and that one in eight men and one in eleven women will die from cancer [2]. Most of the anticancer drugs display non or at best low target selectivity and significant off-target toxicity, which limits their therapeutic value [3]. In this context, nanomedicine research has shown promise in selectively delivering itself to tumors [4]. In general, tumor-targeting with nanomedicines relies on the enhanced permeability and retention effect [5] which is characterized by the higher extravasation of nanosized particles in the tumor regions due to leaky vasculature and defective lymphatic drainage, which would retain the particles longer, leading to enhanced activity [6,7,8,9].

Hydrogel is defined as a tridimensional cross-linked network of hydrophilic or amphiphilic polymer chains able to swell without dissolving in the presence of water [10]. This property of absorbing water is attributed to hydrophilic functional groups present along the polymeric chains, such as -OH, -CON_2_H, and -SO_3_H [11]. Hydrogels are promising materials for different biomedical applications being compatible with many biological environments [11,12]. Microgels are versatile drug delivery systems because of their tunable size from nanometer to micrometer [13], and the swollen network can retain large amounts of solvent [14]. This swelling allows automatic drug loading [15] to preserve drug activity [16], and bioconjugation into the internal network can also be achieved. In the last few decades, core–shell-corona structure hydrogels, with at least one of the layers crosslinked for structural integrity, have emerged as a novel platform for drug delivery [17]. For example, Murphy et al. reported a nanogel drug delivery capable of encapsulating drug chemotypes, displaying targeting ligands on the surface. Nanogels were coated with an integrin to direct the system to tumor tissue and carry distinct chemotype cargoes. These particles exhibited potent activity for the treatment of orthotopic breast and pancreas tumors in mice with taxane-loaded nanogels, which produced a 15-fold improvement in antitumor activity relative to Abraxane by blocking both primary tumor growth and spontaneous metastasis [6].

Among the smart hydrogel materials, NIPAAm has deserved some special attention [18], becoming hydrophobic and insoluble in water above the critical solution temperature (LCST) (32 °C) [19], whereas it is soluble below this value [20]. The addition of ionizable groups into a PNIPAm network provides pH responsiveness, as reported by Magaña et al., and the addition of a carboxyalkyl methacrylamide (CAM5) in response to the ionization of the structure provides pH responsiveness and increases the transition temperature [21,22,23]. Sikhumbuzo et al. reported PNIPAM-bPEI microgels pH- and thermo-dependent polymeric core–shell microgels loaded with Dox. The drug-encapsulated microgels demonstrated drug release profiles that were pH- and thermo-responsive during the in vitro intracellular progressive uptake of Dox into HepG2 cells [24]. Furthermore, Hongrui et al. reported using an antibody-modified prodrug nanoparticle to deliver Dox. The Cd147 monoclonal antibody was bound to the aldehyde group of oxidized dextran (ODEX) and Dox nanoparticles (NPs). Dynamic light scattering was used to evaluate the stability and pH responsiveness in the tumor microenvironment. The in vivo antitumor efficacy and distribution experiments confirmed that CD147-ODEX-Dox NPs could significantly inhibit the growth of a HepG2 tumor in a mouse model [25]. Zhang et al. reported that TAT peptide-modified poly(N-isopropylacrylamide) microgel particles exhibited a thermo-responsive volume expansion ability that copolymerized NIPAM with poly (ethylene glycol) diacrylate and acrylic acid. The PNIAPM microgel particles were largely ingested by A549 cells and mainly located in lysosomes. While the PNIPAM microgel particles did not show significant impacts on cell viability at 37 °C, they caused the strongest cytotoxicity when being cultured at 25 °C for 4 h, suggesting the combinational effect of intracellular volume expansion and drug release on cells [26].

In this context, the aim of this work was to develop a series of temperature- and pH-sensitive microgels capable of adjusting the size-collapse transition to a higher temperature and lower pH, approximating those reported for tumors. This adjustment aims to facilitate the targeted delivery of Dox, thereby reducing its side effects. To achieve this, our group previously demonstrated that incorporating an ionizable group, carboxyalkyl methacrylamide (CAM5), into the PNIPAm network imparts pH responsiveness and raises the transition temperature. By copolymerizing NIPAAm with PEGMA and 10% CAM5, we achieved a lower critical solution temperature (LCST) of 40 °C. These MP 10% CAM5 were characterized and subsequently loaded with Dox, and the release study was performed in different environmental conditions, releasing 25% (pH 7.4/37 °C) and almost 95% (pH 5/42 °C), demonstrating responsiveness to pH and temperature. MP was cytocompatible in mammalian HeLa cell lines. Furthermore, MP 10% CAM5 loaded with Dox (3 µg/mL) were more effective than free Dox, reducing the cell viability to 35% and 75%, respectively, evaluated using an MTT assay in HeLa cell line. This insight could be explained by cellular uptake of the microgels in vitro.

## 2. Results and Discussion

### 2.1. Synthesis and Characterization of Microgels

#### 2.1.1. FTIR

**Figure 1:**
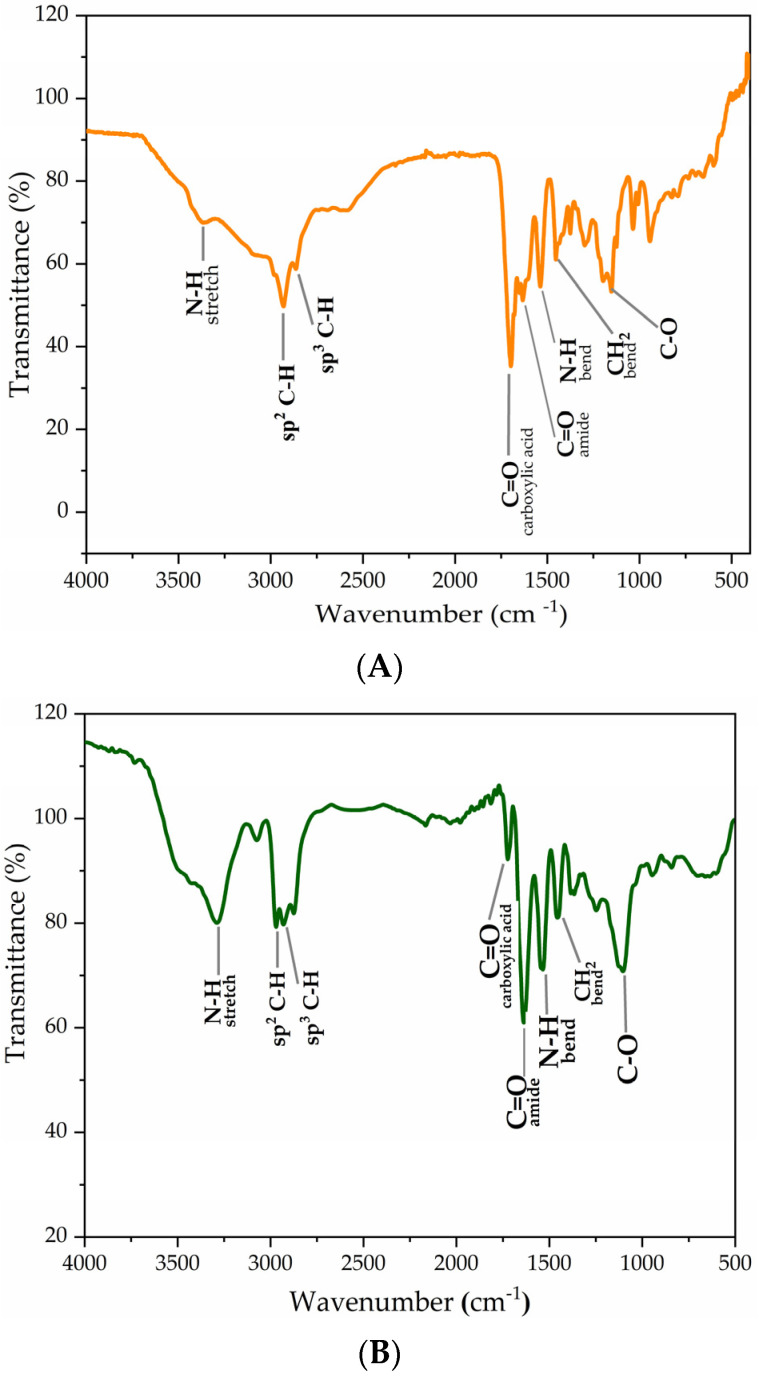

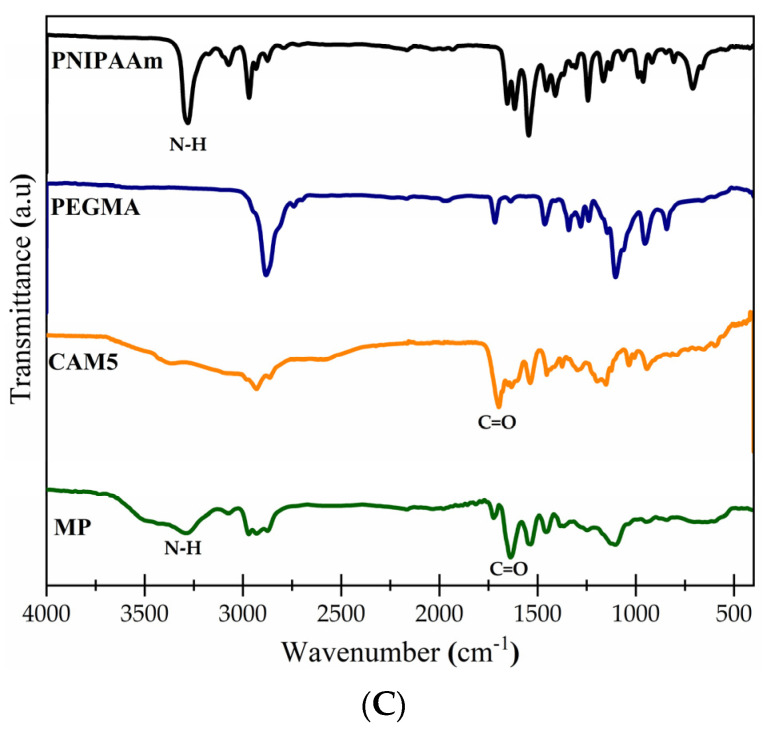
FTIR spectra of (**A**) CAM5 monomer, (**B**) MP, and (**C**) comparative spectra of the monomers used for the synthesis of MP.

The monomers and polymerized microgels (MP) were characterized using Fourier-transform infrared spectroscopy (FTIR) to compare their spectra and confirm successful MP polymerization. FTIR analysis was performed for CAM5 (Figure 1A,C), MP 10% CAM5 (Figure 1B,C), PNIPAAm, and PEGMA (Figure 1C). Characteristic peaks were identified as follows: PNIPAAm: N-H stretching (3288 cm⁻^1^), N-H bending (1535 cm⁻^1^), and C=O (1723 cm⁻^1^). CAM5: N-H stretching (3387 cm⁻^1^), N-H bending (1535 cm⁻^1^), C=O stretching (1723 cm⁻^1^), CH sp^2^ (2930 cm⁻^1^), C=O (carboxylic acid, 1697 cm⁻^1^), C=O (amide, 1638 cm⁻^1^), CH_2_ bending (1459 cm⁻^1^), and C-O (1150 cm⁻^1^). PEGMA: C-H (2900 cm⁻^1^), C-O (1100 cm⁻^1^), and C=O (1700 cm⁻^1^). For MP 10% CAM5, strong absorption bands from both NIPAAm and CAM5 were observed, including N-H stretching (3288 cm⁻^1^), C-H (2970 cm⁻^1^), C=O (carboxylic acid, 1723 cm⁻^1^), C=O (amide, 1637 cm⁻^1^), N-H bending (1535 cm⁻^1^), CH_2_ bending (1456 cm⁻^1^), and C-O (1104 cm⁻^1^). Additionally, Figure 1C shows a shift in the peak near 1630 cm⁻^1^, attributed to the presence of amide functional groups in the monomers, further confirming successful MP polymerization.

#### 2.1.2. Determination of Acid Content

In order to quantify the amount of acid monomer CAM5 loaded in MP, potentiometric titration was used. Microgels with an expected 5, 10, and 15% CAM5 showed 4.5, 10.5, and 16.9%, respectively (Table 1). The determined percentage of CAM5 is higher than the theoretical one, which could be due to a higher interaction of NMAHe with MP precursors.

#### 2.1.3. Particle Size, Zeta Potential, and Transition Temperature

The hydrodynamic size of MP (5, 10, and 15% of CAM5) was determined using dynamic light scattering (DLS) at 25 °C in water, pH 3, pH 5, pH 7, and pH 9. The MP size obtained for 5% (349, 260, 268, 281, 275 nm), 10% (286, 230, 246, 275, 268 nm), and 15% (1265, 1080, 821, 966, 800) of CAM5 in water, pH 3, pH 5, pH 7, and pH 9, respectively, is shown in Table 2. The MP with 5% and 10% CAM5 in pH 3 and 5 exhibited a smaller particle size, demonstrating sensitivity to the pH of the medium. The tendency is to moderately polydisperse, with a polydispersity index (PDI) lower than 0.4 [27]. For 15% MP, larger sizes (1265 nm) and less homogeneity (PDI 0.556) were obtained. These structural changes can be attributed to the increased CAM5 proportion.

**Figure 2:**
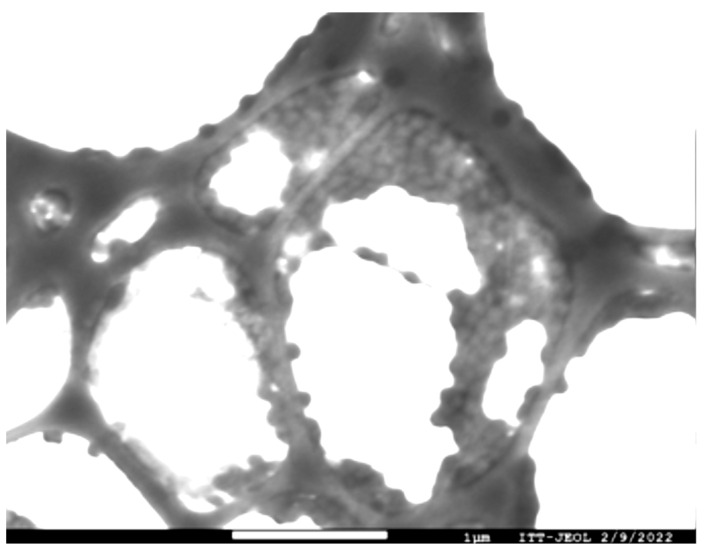
FESEM micrograph of MP 10% CAM5.

The MP (5, 10, and 15% of CAM5) Zeta potential was determined using DLS at 25 °C in water, at pH 3, pH 5, pH 7, and pH 9. The Zeta potential obtained for the MP with various concentrations of CAM5 are as follows: MP 5% CAM5 (−9.99, −3.32, −4.34, −7.32 and −7.56 nm), MP 10% CAM5 (−11.77, −3.52, −13.66, −12.3, −9.17 mV), and MP 15% CAM5 (−4.33, −2.7, −5.16, −16.46, −10.19) in pH 3, pH 5, pH 7, and pH 9, respectively (Table 3). The higher proportion of CAM5 results in a lower Zeta potential due to the acidic nature of the monomer, which increases the anionic charge with an increase in pH above pH 5 [28]. Murillo et al. reported the opposite phenomena, with the size decreasing and a bigger zeta potential when hyperbranched alkyd resin (HAR) was polymerized with increasing proportions of methyl methacrylate, butyl acrylate, and acrylic acid. The resulting particles have their particle size increased with the content of HAR, but have lower colloidal stability, critical deformation, zeta potential, thermal stability, and hardness [29]. Our work group previously reported temperature- and pH-sensitive core–shell nano/microgels with a crosslinker (core) of N-isopropylacrylamide and increasing proportions (5, 10, and 15%) of methacryloylamido hexanoic acid (CAM5) or the salt form of CAM5. The variation in the % of acid CAM5 resulted in a bigger size particle (microgels) and an adjustment to biological temperature LCST. Furthermore, with the CAM5 salt form, we get smaller (nanogels) particles, but the LCST did not change [28]. Our results showed, as seen in Figure 2, that adding PEGMA results in smaller particles, with LCST adjusted to the tumor microenvironment.

These suggest colloidal stability and cytocompatibility, because there is less risk of destabilizing cellular membranes while keeping the values of Z potential in the range of −30 to 30 mV [27].

With these previous results of homogeneous particle size and low zeta potential values, we decided to continue characterizing MP 10% CAM5. To corroborate the particle size and morphology, they were characterized by field emission electron microscopy (FESEM). The micrography (Figure 2) showed an average size of 121 nm, which is smaller than the size determined using DLS because samples are dehydrated for FESEM microscopy [30]. Micrographs corroborate the homogeneity of the synthesis and spherical shape of MP 10% CAM5.

#### 2.1.4. Particle Size in Respect to Temperature

Temperature sensitivity is the main characteristic of N-isopropylacrylamide microgels relevant to biomedical applications [3]. Microparticles of NIPAAm with amphiphilic monomers displace the size transition temperature from 32 °C towards higher temperatures [28]. Cancer tumors have a temperature niche higher than the human body, and for these reasons, the aim of this study is to increase the LCT of NIPAAm for responsive tumor drug delivery [30].

**Figure 3:**
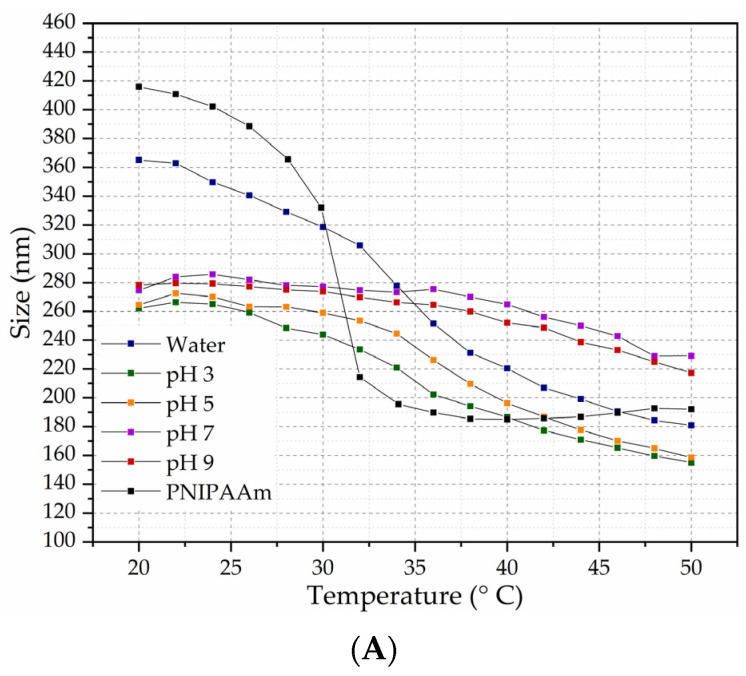

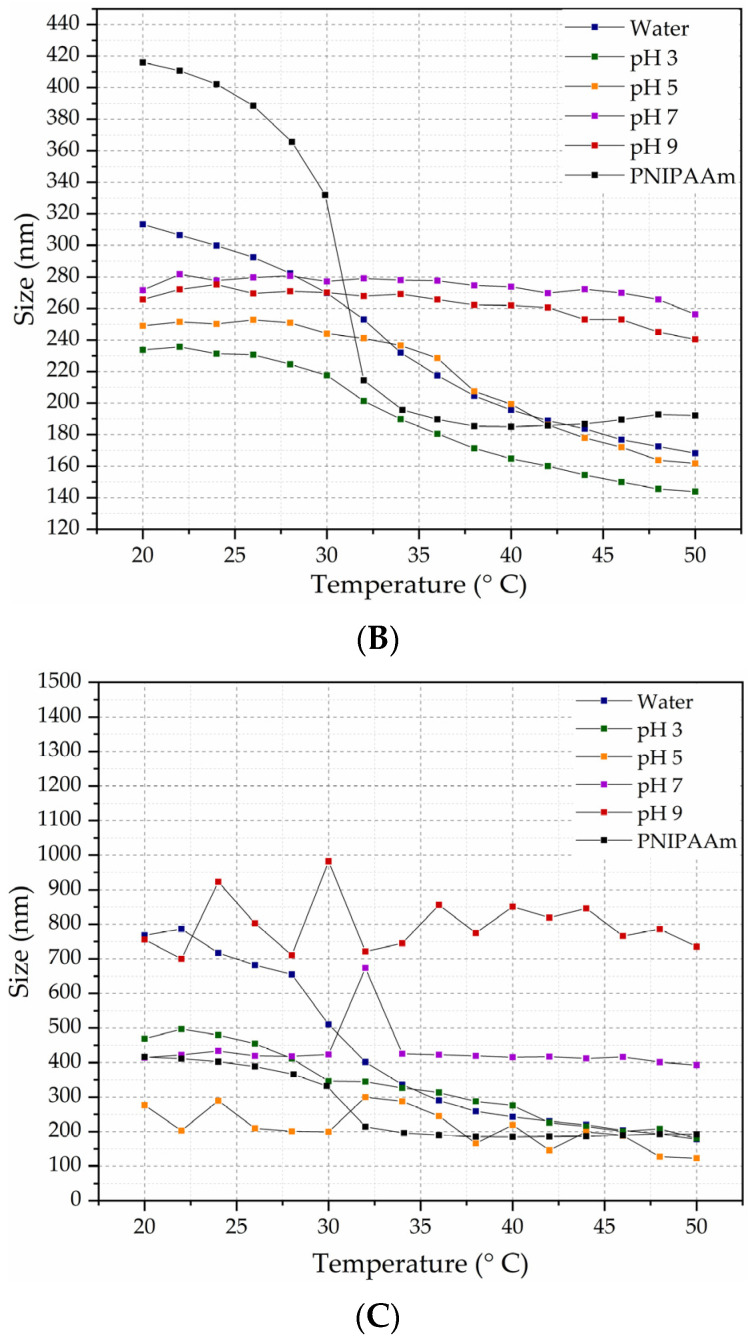
Temperature trends of (**A**) MP 5% CAM5, (**B**) MP 10% CAM5 and (**C**) MP 15% CAM5 in water, pH 3, pH 5, pH 7, and pH 9.

To determine the transition temperature of 1% MP with 5, 10, and 15% CAM5, the size-temperature analysis was performed from 20 to 50 °C in pH 3, 5, 7, and 9. In Figure 3, the results are presented as size-temperature comparative graphics. As shown in Figure 3A,B, with the well-known NIPAAm size transition at 32 °C from 420 to 190 nm, there is no LCT with MP (5% and 10% of CAM5) in pH 7. However, there is a size reduction from 260 nm (30 °C) to 200 nm (40 °C) for MP 5% CAM5 and from 240 (30 °C) to 200 nm (40 °C) for MP 10% of CAM5 in pH 5.

To determine the transition size-temperature, the first derivatives of size versus temperature were calculated for 5, 10, and 15% CAM5 in water, at 3, 5, 7, and 9 pH, as shown in Table 4. Incorporating 10% and 15% of the CAM5, the size versus temperature plot did not show a consistent LCT in pH 7. On the other hand, incorporating 5 and 10% of the CAM5 increased by 10 °C the LCT of NIPAAm, from 32 to 42 °C, in pH 5, in accordance with Figure 3. MP 10% CAM5 was selected for further analysis because features of responsiveness to pH and temperature results are promising for biomedical applications, such as tumor treatment, where the temperature rises compared to the surrounding tissues as a result of the increased abnormal metabolic activity [31].

We previously showed microgels with increasing proportions (5, 10, and 15%) of CAM5 or the salt form. Microgels resulted in an adjusted transition temperature of 38 °C and 1194 nm, and the microgels containing the salt form exhibited a reduced particle size of 357 nm but the transition temperature was 32 °C [28]. With these previous results, we hypothesize that the ionized monomers do not interact with the NIPAAm, but act as surfactants distributed on the surface of the microgel. For this reason, we decided to incorporate PEGMA in this new formulation, and the resulting microgels showed both smaller sizes and adjusted transition temperatures as we expected.

### 2.2. Drug Loading

**Figure 4:**
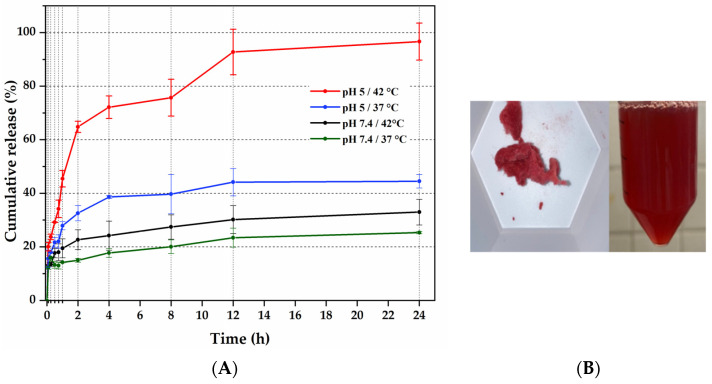
(**A**) Cumulative release of Dox at different conditions, (**B**) Dox-MP 10% CAM5.

The MP 10% CAM5 loaded with Dox was recovered using centrifugation and freeze-dried. The drug loading and encapsulation efficiency was calculated by subtracting the Dox remnant supernatant. This confirms the Dox loading into MP 10% CAM5 by the presence of the red color (Figure 4B). The drug loading and encapsulation efficiency were 13% and 87%, respectively.

### 2.3. Drug Release Study

The Dox release study was performed by placing microgels in release media at pH 5 or 7.4 and 37 °C or 42 °C (Figure 4A). The Dox released was quantified by UV-Vis_485 nm_. The percentage of drug released up to 24 h at pH 7.4 was 24% at 37 °C and 32% at 42 °C, at pH 5 was 44% at 37 °C and 96% at 42 °C. This behavior may be due to the sensitivity of MP 10% CAM5 to the lower pH release medium, which has been shown to decrease the size and increase transition temperature, as previously reported [28].

Our work group previously reported temperature- and pH-sensitive core–shell nanogels with a crosslinker (core) of N-isopropylacrylamide and 2-methacryloyloxy benzoic acid with a stabilizer (shell) of poly (ethylene glycol) methyl ether methacrylate (EGDMA), capable of load up to 34% (wt%) [32]. Ye et al. reported 30–40 nm spherical doxorubicin nanoparticles coated with chitosan and as biorecognition element folic acid, with a 10.73% and 65% doxorubicin loading efficiency and release, respectively. In HepG2 culture, nanoparticles showed an improvement in decreased cell viability (approximately 60%) compared with free doxorubicin (approximately 40%) [33].

### 2.4. Cell Viability

**Figure 5:**
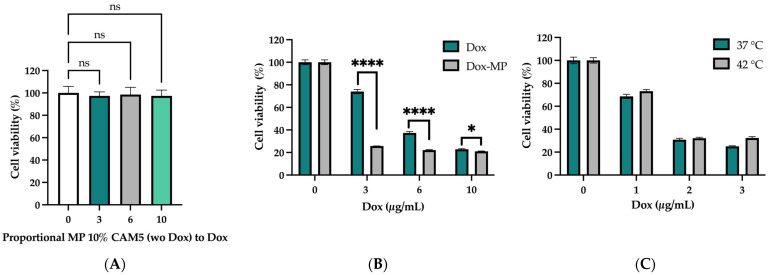
In vitro cytotoxicity in HeLa cell line of (**A**) MP 10% CAM5 (without Dox), (**B**) Dox vs. Dox-MP 10% CAM5 and, (**C**) Dox-MP 10% CAM5 (37 vs. 42 °C). Data are presented as means ± SD (*n* = 5). * *p* 0.0366, **** *p* < 0.0001 vs. Dox. No statistically differences were detected among treating cells at different temperatures (ns).

The indirect cytotoxicity of Dox-MP 10% CAM5 and MP 10% CAM5 was evaluated in HeLa cells using the MTT assay, as a measurement of cell viability in response to micromaterial exposition. Cell cultures were treated with the proportional amount of MP 10% CAM5 (without Dox) contained in the proposed Dox formulations Dox-MP 10% CAM5 (3, 6, and 10 µg/mL). The MP 10% CAM5 did not affect HeLa cell culture viability (Figure 5A). Formulation Dox-MP 10% CAM5 showed dose-dependent cytotoxicity (Figure 5B). At higher concentrations of Dox (10 µg/mL), free Dox and Dox-MP 10% CAM5 decrease to 20% the HeLa cell viability. At lower concentrations of Dox, with a 3 and 6 µg/mL formulation, Dox-MP 10% CAM5 exhibited a better drug cytotoxicity capability than free Dox, which decreased to 37 and 74%, respectively. These findings demonstrate that Dox encapsulation in MP 10% CAM5 significantly improves its cytotoxic capacity compared to Dox. This improvement is particularly evident with 3 and 6 µg/mL concentrations, where Dox-MP 10% CAM5 has a pronounced reduction in cell viability. These findings demonstrate that MP 10% CAM5 could efficiently reach monolayer tumor culture cells. A possible explanation for this may be attributed to the internalization of the Dox-MP 10% CAM5 into the cells. Zhang et al. reported the cell uptake of PNIPAM microgels by lung adenocarcinoma (A549) cells, which were largely ingested and mainly located in lysosomes [26]. This previous report on cellular uptake and subcellular distribution in lysosomes allows us to hypothesize that Dox-MP 10% CAM5 may accumulate in lysosomes, where the lower pH could favor Dox release. This hypothesis should be addressed in the future.

To compare whether there is a response to temperature increase in vitro, cells were exposed to Dox-MP 10% CAM5 (3, 6, and 10 µg/mL) at 42 °C for 24 h (Figure 5C). However, there was no significant decrease in cell viability. This may be because in this experiment, the two proposed environmental conditions, low pH and high temperature, do not occur at the same time, since a tumor could be found.

## 3. Conclusions

Microgels were synthesized from the copolymerization of NIPAAm, PEGMA, and CAM5 (5, 10, and 15%). The synthesized MP showed homogeneous particle sizes, with PdI lower than 0.4. MP exhibiting responsiveness to pH changes. In the same way, it was demonstrated that copolymerization with CAM5 increases the transition temperature of NIPAAm microgels from 36 to 42 °C for promising treatments of pathologies that raise the temperature of regions of the human body, such as cancer.

The cytotoxic effect of Dox was greater when loaded into MP 10% CAM5; therefore, lower amounts of Dox are needed to reach cellular cytotoxic levels, demonstrating the cytotoxic potency of Dox-MP 10% CAM5 on tumor cells. These phenomena can be explained by the cellular uptake of Dox-MP 10% CAM5 into the cytoplasm and preferentially into lysosomes, where low pH increases Dox release and, therefore, increases intracellular Dox concentration. These results indicate that microgels can respond to the temperature and pH of the tumor microenvironment, increasing the concentration of the drug available in the tumor, thereby helping to reduce the unwanted effects of anticancer drugs such as Dox, and allowing effective treatment with a lower dose of Dox needed for cancer treatment.

The drug delivery system designed in this project has potential application in tumor microenvironments with sensitivity to higher temperatures and lower pH than the healthy human body.

## 4. Materials and Methods

### 4.1. Materials

The N-isopropylacrylamide (NIPAAm, ≥97%, MERCK, Rahway, NJ, USA) was purified via recrystallization from hexane. Ethylene Glycol Dimethacrylate (EGDMA, MERCK, Naucalpan, México) was purified using a column of hydroquinone remover, Ammonium persulfate (APS, MERCK, México), Poly (ethylene glycol) methyl ether methacrylate (PEGMA, Mn 1100, MERCK USA), Hexane (Fermont México, ≥98.5%), doxorubicin (≥95.0%, TCI America, Portland, OR, USA), Dulbecco’s Modified Eagle medium (DMEM, Biowest, Phosphate Buffer Saline (PBS, Gibco, MERCK, México), Fetal Bovine Serum (FBS, Gibco, MERCK México), and a cell proliferation kit (MTT, Roche applied science USA, Indianapolis, IN, USA).

### 4.2. Synthesis of Microgels

#### 4.2.1. Synthesis of Amphiphilic Monomer: 6-Methacryloylamidohexanoic Acid (CAM5)

**Figure 6:**
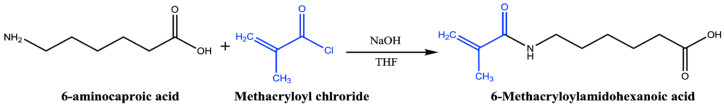
Scheme of amphiphilic monomer CAM5 synthesis.

The synthesis of amphiphilic acid, 6-methacryloylamidohexanoic acid (CAM5), was performed using the Schotten–Bauman method from 6-aminocapropic acid and methacryloyl chloride (Figure 6), as reported by Magaña et al. [21] Briefly, 6-aminocaproic acid was dissolved in a solution of NaOH 1 M with Tetrahydrofuran, and methacryloyl chloride was added drop by drop. Then the resulting solution was acidified with HCl to pH 3, and finally, the monomer was extracted with ethylic ether and purified. The resulting product was characterized by FTIR-ATR and ^1^H NMR.

#### 4.2.2. Synthesis of Microgels

**Figure 7:**
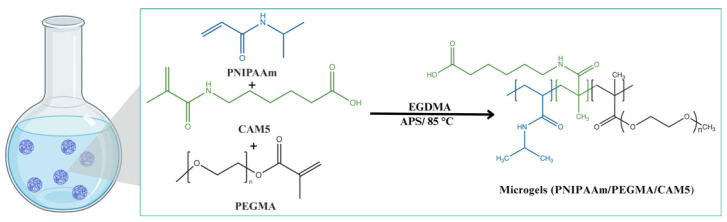
Schematic representation of the route synthesis of the microgels MP.

The microgels (MP) were prepared using the procedure of dispersion polymerization as follows: 0.35 g of NIPAAm, 0.15 g of PEGMA, CAM5 (5, 10 or 15% mol), and 5% mol of crosslinker EGDMA (Figure 7) were acquired, and then dissolved in 50 milliliters of distilled water in a single-necked flask, stirring for 30 min. The oxygen was removed via Nitrogen purging. Then, the mixture was heated to 85 °C to initiate the reaction, and 2% mol of APS as initiator was added to the solution and stirred for 45 min. The flask was placed in a water/ice bath to stop the polymerization process [28]. The solution containing MP was purified by dialysis tubing (12–14 kDa) for 7 days. The microgels were recovered by freeze-drying.

### 4.3. Characterization of Microgels

#### 4.3.1. Infrared Spectra

The infrared spectra were determined using a Fourier Transform Infrared spectrophotometer (FT-IR) Thermo Scientific™ Nicolet™ iS™5 with an attenuated total reflectance (ATR) module, using the direct method at room temperature. Samples of freeze-dried microgels were analyzed [28].

#### 4.3.2. Particle Size

The hydrodynamic diameter of the microgels was determined using dynamic light scattering (DLS) (Zetasizer Nano NS, Malvern instruments). Samples were prepared by disssolving 1 mg of freeze-dried microgels (MP) in 1 mL of water. To assess the influence of pH, MP were dispersed in buffer solutions with pH values ranging from 3 to 9.

Field emission scanning electron microscopy (FESEM) corroborated the MP size and morphology. The sample was prepared by placing a 0.2% dispersion of MP 10% CAM5 on a copper-carbon grid and then fixed with a 1% uranyl acetate solution [33].

#### 4.3.3. Transition Temperature

The effect of the temperature on the size of the MP was evaluated using DLS (Zetasizer Nano NS, Malvern instruments) with the temperature trend method over a range of 20 to 50 °C. Dispersions of freeze-dried MP (1 mg/mL) were prepared in water and in buffer at different pH values to determine the impact on the transition temperature [28].

#### 4.3.4. Z-Potential

The Zeta potential of MP was measured using DLS (Zetasizer Nano NS, Malvern instruments) to determine the surface charge of the particles. Samples were prepared by diluting 10 μL of a 1 mg/mL MP dispersion in 1 mL of buffer with the pH values ranging from 3 to 9 [28].

#### 4.3.5. Determination of the Acid Content

The content of the copolymerized CAM5 was determined using acid-base titration. First, 50 mg of the freeze-dried MP was dispersed in 5 mL of distilled water. To guarantee the complete ionization of the carboxylic acid groups, 0.05 M NaOH was added up to pH 11. Then, a necessary volume of 0.05 M HCl was added until pH 3 [28].

### 4.4. Drug Loading Study

Doxorubicin (Dox) was loaded in MP, and drug loading capacity and encapsulation efficiency were determined. Briefly, a known amount of freeze-dried MP 10% CAM5 (30 mg) was dispersed in 8 mL of water. Then, a molar equivalent solution of 0.01 M NaOH was added to ionize the acidic groups. Subsequently, a solution of Dox hydrochloride (4.5 mg/1.25 mL of water) was added drop by drop to promote an ionic interaction between the groups. MP 10% CAM5 was allowed to interact with the Dox solution at 20 °C with constant stirring overnight, looking for a hydrophobic or hydrogen-bridging interaction. To collect the Dox-loaded microgels, the sample was centrifuged at 9500 rpm at 20 °C for 60 min to remove the free Dox present in the supernatant.

The quantification of Dox was performed with a calibration curve using a stock solution composed of DMA: H_2_O (1:1) pH 3 with a concentration of 1 mg/mL of Dox, and samples were taken at different dilutions to obtain concentrations in μg/mL dissolved in DMA: H_2_O (1:1) pH 3. The drug process control analyses were performed using the UV-Vis spectrophotometer at 485 nm wavelength and the blank solutions of DMA: H_2_O (1:1) pH 3 [34].

To calculate the loading efficiency and loading capacity, we dispersed 1 mg of Dox-MP 10% CAM5 in a mixture of DMA:H_2_O (1:1) adjusted pH 3 at a temperature of 4 °C with constant agitation in a water circulator for 5 h. Afterwards, it was analyzed using UV-Vis to obtain the concentration of the drug contained. The loading efficiency and loading percentage were calculated according to the formulas 1 and 2 [34]:

(1) $$\%\;Entrapment\;Efficiency\left(EE\right)=\frac{Initial\;Dox-Supernatant\;Dox}{Inicial\;Dox}*100$$

(2) $$\%\;Drug\;Loaded\;(DL)=\frac{Initial\;Dox-Supernatant\;Dox}{MP\;weight}*100$$

### 4.5. In-Vitro Drug Release

The drug release study was performed using the dialysis membrane method. Briefly, a known amount of Drug-MP 10% CAM5 that contains 1 mg of Dox was placed inside a dialysis membrane. This assay was performed by simulating both physiological conditions (pH 7.4) and acidic conditions (pH 5) at temperatures of 37 and 42 °C. It was carried out in an oscillating water bath with temperature control at 50 rpm. Samples of the medium were taken at predefined time periods (0.25, 0.50, 0.75, 1, 2, 4, 8, 12, and 24 h [34]).

#### Quantification of Dox

A calibration curve was performed using a stock solution at a concentration of 1 mg/mL of Dox in water and a series of samples with different dilutions to obtain concentrations on the µg/mL scale dissolved in distilled water. It was quantified using UV-Vis spectrometry at 485 nm wavelength [35].

### 4.6. Cell Viability

Cell viability was evaluated using the MTT assay. Briefly, $7.5\;{\times 10}^{4}$ cells of Hela cells were exposed to Dox at concentrations of 3, 6 and 10 μg/mL, as well as to proportional amounts of Dox-MP 10% CAM5 and with MP 10% CAM5. The assay was conducted at 37 °C and in a parallel experiment, cells were incubated at 42 °C for 24 h to assess potential temperature-dependent sensitivity [36].

## Figures and Tables

**Table 1:** Content of CAM5 in MP using potentiometric titration.

**% CAM5 Theoretical**	5	10	15
**% CAM5 Obtained**	4.6	10.5	16.9

**Table 2:** Size of MP with 5, 10, or 15% of CAM5 in different dispersion media, obtained by DLS.

Dispersion Media	Data	5%	10%	15%
Water	Size (nm)	349	286	1265
	±SD	5.4	0.7	345
	PDI	0.129	0.099	0.556
pH 3	Size	260 ****	230 *	1080
	±SD	7.6	0.7	373
	PDI	0.112	0.116	0.52
pH 5	Size (nm)	268 ****	246	821
	±SD	1	1	105
	PDI	0.106	0.076	0.425
pH 7	Size (nm)	281 ****	275	966
	±SD	0.6	31.5	185
	PDI	0.104	0.102	0.393
pH 9	Size (nm)	275.8 ****	268.9	800.7
	±SD	2.8	0.8	375
	PDI	0.086	0.089	0.51

Data are presented as mean ± SD (*n* = 3). * *p* < 0.0290, **** *p* < 0.0001, vs. water.

**Table 3:** Zeta potential of MP with different ratios of CAM5.

	Content of CAM5		
Dispersion media	5%	10%	15%
	Zeta Potential (mV) *(±SD)*		
water	−9.99 (±0.4)	−11.77 (±0.6)	−4.33 (±0.2)
pH 3	−3.32 (±0.5)	−3.52 (±0.95)	−2.7 (±0.9)
pH 5	−4.34 (±1)	−13.66 (±2.1)	−5.16 (±0.5)
pH 7	−7.32 (±0.4)	−12.3 (±1.85)	−16.46 (±2)
pH 9	−7.56 (±4)	−9.17 (±0.3)	−10.19 (±0.7)

**Table 4:** Transition temperatures of MP with 5, 10, and 15% of CAM5 at different dispersion media.

Dispersion Media	Content of CAM5		
	5%	10%	15%
	Temperature (°C)		
Water	42	38	36
pH 3	38	36	*
pH 5	42	42	*
pH 7	34	40	*
pH 9	42	42	*

* The temperature trends were not able to calculate the transition.

## Data Availability

The original contributions presented in the study are included in the article. Further inquiries can be directed to the corresponding author.
